# Portal thrombosis after surgical treatment of schistosomatic portal hypertension

**DOI:** 10.1590/1806-9282.20231689

**Published:** 2024-07-19

**Authors:** Leonardo de Souza Vasconcellos, Vivian Resende, João Bernardo Sancio Rocha Rodrigues, Andy Petroianu

**Affiliations:** 1 Universidade Federal de Minas Gerais, School of Medicine – Belo Horizonte (MG), Brazil.

**Keywords:** Schistosomiasis mansoni, Portal hypertension, Surgical procedure, Splenectomy, Postoperative complications, Portal vein, Thrombosis

## Abstract

**OBJECTIVE:**

Several studies have investigated the correlation between the effects of different surgical treatments and laboratory exams for schistosomal portal hypertension, especially concerning portal system thrombosis. The etiopathogenic factors of this thrombosis are not fully understood. In this study, the correlation between surgical treatment for schistosomal portal hypertension and the occurrence of postoperative portal system thrombosis was investigated.

**METHODS:**

A total of 61 patients who underwent surgical treatment for schistosomal portal hypertension were distributed into four groups: Patients in Group 1 (n=12) underwent portal variceal disconnection associated with splenic artery ligation and spleen preservation. Patients in Group 2 (n=20) underwent portal variceal disconnection and total splenectomy. Patients in Group 3 (n=20) underwent portal variceal disconnection with subtotal splenectomy, preserving the upper splenic pole supplied by the splenogastric vessels. Patients in Group 4 (n=9) underwent portal variceal disconnection with total splenectomy and autogenous splenic implants on the greater omentum. Late postoperative portal vein thrombosis was diagnosed using Doppler ultrasound.

**RESULTS:**

Over the 10-year follow-up, portal vein thrombosis occurred in 26 operated patients (42.6%), with no significant difference observed among the four surgical groups (p=0.217). Most of the thrombi only partially occluded the portal system veins. All the patients presented with a thrombus inside the portal vein. There was no difference in hematological and biochemical tests between groups with or without portal vein thrombosis.

**CONCLUSIONS:**

Portal vein thrombosis is often observed in the late postoperative period, irrespective of the surgical treatment employed, and is not associated with patient characteristics or any hematological and biochemical tests.

## INTRODUCTION

Schistosomiasis is an endemic disease in 52 countries, ­including those in South America, the Caribbean, Africa, and Asia. According to the World Health Organization, more than 200 million people are infected by one of the *Schistosoma* ­species (*Schistosoma mansoni*, *Schistosoma japonicum*, *Schistosoma ­haematobium*, and *Schistosoma mekongi*), and in 10% of them, the disease progresses to the ­hepatosplenic form^1^. ­Pre-sinusoidal hepatosplenic ­schistosomal portal hypertension is a primary complication of *S. mansoni* ^2^.

In advanced stages, the rupture of gastroesophageal ­varices is frequent, and its morbimortality varies according to the ­hemorrhagic intensity^3^. Given its benign nature that ­preserves the liver ­function, the treatment of variceal bleeding and its ­prevention must be ­effective, minimizing the risk of ­recurrence^4^. A well-performed portal ­variceal disconnection (PVD) with partial or subtotal ­splenectomy appears to be the most ­effective long-term treatment for reducing the ­pressure in the ­esophagogastric veins and ­alleviating ­splenomegaly ­discomfort without ­compromising the spleen defensive ­function^5^. Despite the absence of a perfect ­operation for treating complicated portal hypertension, various techniques have been applied based on the preferences of the ­surgeon. However, there are no significant differences among the results of these techniques, and they ­generally provide ­satisfactory outcomes despite potential adversities^4,5^.

The most common postoperative complication is portal vein thrombosis, occurring in up to half of all patients, and is ­typically transient^6-8^. The size and formation of the ­thrombus are ­unpredictable, and it is always spontaneous, transient, and ­recurrent^9,10^. Although the splenoportal thrombus may extend to other veins, such as the superior and inferior mesenteric veins and the left gastric vein, no motility or absorptive or metabolic digestive disorders have been associated with this event. Mesenteric ­ischemia with intestinal necrosis occurs in less than 1% of all cases^10,11^.

The purpose of this study was to investigate the ­correlation between four different types of surgical ­treatments for ­schistosomal portal hypertension and postoperative ­portal ­system thrombosis, as well as to assess the impact of ­hematological and ­biochemical blood tests on this association.

## METHODS

### Study design and selection of patients

This research is part of a series of studies related to portal ­hypertension treatment^4,8,12-14^. This prospective study involved 61 ­consecutive adult patients who were followed up over a 10-year period at the Clinical Hospital of Universidade Federal de Minas Gerais, Brazil. All patients had severe esophageal and gastric ­variceal bleeding resulting from hepatosplenic *S. ­mansoni* ­associated with splenomegaly^3,4^. Before the surgical procedure, they underwent treatment with oxamniquine or ­praziquantel. Patients requiring emergency surgical procedures were not included^8^.

All patients gave their informed consent for inclusion in the study. The study was conducted in accordance with the Declaration of Helsinki, and the protocol was approved by the Ethics Committee at the Universidade Federal de Minas Gerais, Brazil, under registration number ETIC 006/08.

## SURGICAL PROCEDURES

The PVD was performed by ligating all veins of the gastric lesser curvature and posterior wall, including the left gastric vein, as well as the veins surrounding the abdominal ­esophagus. The anterior wall of the stomach was ­longitudinally opened, and all the major gastric varices were ligated with running sutures up to the lower esophagus, using polyglycolic acid thread. The gastrotomy was closed in two layers with absorbable running sutures^4,5^. A liver biopsy was performed during the surgical procedure to confirm schistosomiasis and exclude other disorders^4,13-15^.

**Figure 1:**
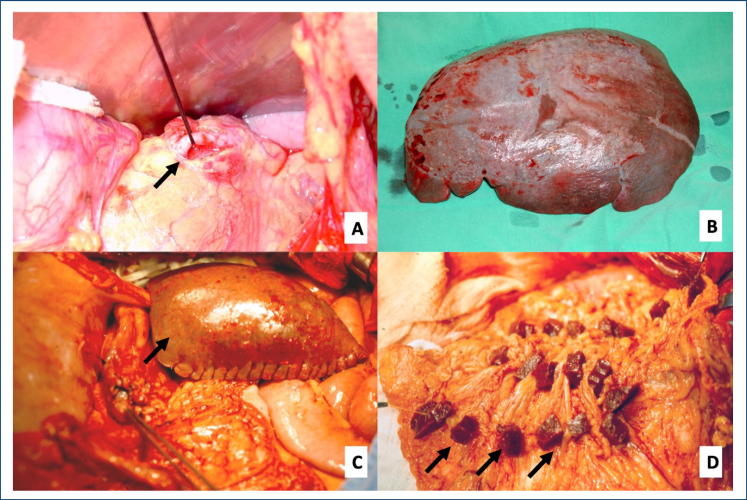
Surgical procedures for the treatment of schistosomal portal hypertension. (A) Tying the splenic artery (arrow). (B) Total splenectomy. (C) Subtotal splenectomy with preservation of the upper splenic pole (arrow). (D) Total splenectomy followed by autogenous splenic implants (arrows) on the greater omentum

The patients were distributed into four groups based on the four surgical procedures recommended in the ­literature for ­treating portal hypertension without ­vascular shunt^4-7,11,16^ (Figure 1):

- Group 1 (n=12): PVD associated with splenic artery ­ligature and spleen preservation;
- Group 2 (n=20): PVD associated with total splenectomy;
- Group 3 (n=20): PVD associated with subtotal ­splenectomy, maintaining the upper splenic pole ­supplied by the ­splenogastric vessels;
- Group 4 (n=9): PVD associated with total ­splenectomy and 20 autogenous splenic implants on the greater omentum.

## VARIABLES AND OUTCOMES

The clinicodemographic and laboratory exams considered in this study included: age, sex, complete blood count, prothrombin activity, international standardized ratio (INR), urea, creatinine, glucose, total and fractionated cholesterol, triglycerides, albumin, globulins, alkaline phosphatase, ­gamma-glutamyltransferase, aminotransferases, and bilirubins^13,14^. Throughout the 10-year follow-up period, all patients underwent annual laboratory exams and a two-dimensional Doppler ultrasound study, or as needed. The primary outcome aimed to investigate ­thrombosis in the portal venous system, including portal, splenic, superior mesenteric, inferior mesenteric, and left gastric veins. Thrombi were classified as partial, cavernous (undergoing recanalization), and total^7,15^. The second outcome was to verify the association between the laboratory variables and the presence of a ­thrombus in the portal venous system.

## STATISTICAL ANALYSES

The power calculation accepted samples higher than eight patients in each group. Descriptive statistics were expressed as median values (interquartile range [IQR]) for continuous ­measures and frequency (percentage) for categorical measures. The incidence of thrombosis was compared using the ­chi-squared test or Fisher's exact test, as appropriate. Laboratory test results were compared by analyzing patients with and without ­thrombosis, using Student's t-test, with a significance of p<0.05.

## RESULTS

The vast majority of patients were male (n=40, 65.6%) with an average age of 43±11 years, while 21 were female (34.4%), with an average age of 45±12 years. Cirrhosis or liver tumors were not recorded during the 10-year follow-up. All patients ­exhibited fibrotic splenomegaly and hepatic fibrosis ­characteristic of *S. mansoni*.

Portal system thrombosis was identified in 26 cases (42.6%) with no difference in age, sex, surgical procedure, or ­post-operative laboratory exams (Table 1). Partial thrombus was found in 20 (77%) patients, while cavernous thrombus was observed only in 6 (23%) patients. Most of the thrombi only partially occluded the portal system veins; no total portal vein obstruction was found in this series. All patients with thrombosis presented a thrombus inside the portal vein, either isolated (n=13, 50.0%) or associated with other veins of the portal system: splenic vein (n=10, 38.5%), superior mesenteric vein (n=2, 7.7%), and inferior mesenteric vein (n=1, 3.8%) (Table 1).

**Table 1:** Presence of thrombus in the portal vein system during the late postoperative period of 61 patients with schistosomal portal hypertension.

		Group 1(n=12)	Group 2(n=20)	Group 3(n=20)	Group 4(n=9)	Total(n=61)
Portal vein system thrombus*						
	Negative	7 (58.3%)	9 (45%)	15 (75%)	4 (44.4%)	35 (57.4%)
	Positive	5 (41.7%)	11 (55%)	5 (25%)	5 (55.6%)	26 (42.6%)*
Thrombus classification with percentages related only to the positive cases						
	Partial	4 (80%)	8 (72.7%)	4 (80%)	4 (80%)	20 (77%)
	Cavernous	1 (20%)	3 (27.3%)	1 (20%)	1 (20%)	6 (23%)
Thrombus location with percentages related only to the positive cases						
	Portal vein	5 (100%)	11 (100%)	5 (100%)	5 (100%)	26 (100%)
	Splenic vein	1 (20%)	4 (36.4%)	3 (60%)	2 (40%)	10 (38.5%)
	Superior mesenteric vein	0	1 (9.1%)	0	1 (20%)	2 (7.7%)
	Inferior mesenteric vein	0	1 (9.1%)	0	0	1 (3.8%)

Data are expressed as n (%). Group 1 (n=12): portal variceal disconnection associated with splenic artery ligature and spleen preservation; Group 2 (n=20): portal variceal disconnection associated with total splenectomy; Group 3 (n=20): portal variceal disconnection associated with subtotal splenectomy, maintaining the upper splenic pole supplied by the splenogastric vessels; Group 4 (n=9): portal variceal disconnection associated with total splenectomy and 20 autogenous splenic implants on the greater omentum.

* Portal thrombosis: p=0.217.

No differences were observed in hematological and ­biochemical results when comparing patients of all groups with and without thrombosis during the 10-year follow-up (Table 2).

**Table 2:** Association between portal vein thrombosis and laboratory exams (mean ± standard deviation of the mean) in 61 patients with schistosomal portal hypertension 1 year after the surgical procedure.

Exams	Portal thrombosis		p-value
	Positive	Negative	
Red blood cells (×10^6^/mm^3^)	4.72±0.60	4.71±0.47	0.815
Hemoglobin (g/dL)	12.7±1.7	13.6±1.8	0.104
Hematocrit (%)	38.0±5.3	40.5±4.7	0.112
Total white blood cells (cells/mm^3^)	6570±2347	7085±2650	0.426
Eosinophils (%)	7±7	5±4	0.213
Platelets (×10^3^/mm^3^)	302±137	254±136	0.177
Prothrombin activity (%)	74±15	80±16	0.156
International normalized ratio–INR	1.21±0.18	1.17±0.16	0.308
Albumin (mg/dL)	4.1±0.5	4.2±0.4	0.353
Globulins (mg/dL)	3.4±0.3	3.3±0.4	0.149
Alkaline phosphatase (U/l)	117±37	111±45	0.495
Glutamyl transferase range (U/l)	80±29	68±35	0.128
Aspartate aminotransferase (U/l)	40±8	39±10	0.731
Alanine aminotransferase (U/l)	40±8	36±12	0.180
Direct bilirubin (mg/dL)	0.2±0.1	0.2±0.2	0.940
Indirect bilirubin (mg/dL)	0.6±0.3	0.7±0.3	0.360
Total cholesterol (mg/dL)	179±26	186±31	0.373
High density cholesterol (mg/dL)	47±8	50±10	0.253
Low density cholesterol (mg/dL)	110±25	117±30	0.327
Very low density cholesterol (mg/dL)	22±7	19±6	0.122
Triglycerides (mg/dL)	108±37	95±30	0.136
Glucose (mg/dL)	89±9	86±7	0.256
Urea (mg/dL)	28±9	30±11	0.301
Creatinine (mg/dL)	0.8±0.2	0.7±0.2	0.346

## DISCUSSION

Schistosomiasis has become a focal point in various lines of research within the literature. This disease causes ­peritoneal inflammation with several consequences, including ­chronic iliac pain due to abdominal and pelvic adhesions^15^. Therefore, the accuracy of treatment is important for practical clinics. The increased availability of diagnostic resources, notably Doppler ultrasonography, has enabled more ­effective follow-up of patients treated for schistosomiasis. These resources have disclosed the high incidence of thrombosis in the ­portal system during the postoperative period as an additional ­post-operative adverse event of the surgical treatment for complicated ­schistosomal portal hypertension^6^. According to Widman et al., these rates range from 19% after PVD with total splenectomy to 50%, after distal splenorenal shunt^6^. Cleva et al. observed an ­incidence of up to 55% of post-­portal-variceal ­disconnection thrombosis^7^.

The first report of portal thrombosis was in 1956, with the necropsy of a cirrhotic patient^17^. In schistosomiasis, ­portal ­thrombosis was described by Bogliolo^17^. Since the 1970s, with the advent of ultrasound, which has been ­associated with Doppler in the 1980s, many reports of postoperative portal ­thrombosis have been published^18-21^, generally ­without clinical consequences^22-25^. In this study, the prevalence of ­thrombosis was 42.6% during the 10-year follow-up period, and no patient ­presented ­symptoms related to this event. The ­assessment of ­portal thrombosis took place during the yearly ­general ­examination of each patient. Some patients ­experienced more than one ­episode of ­portal thrombosis during the total follow-up, and typically, the thrombi resolved within ­different periods, with none ­persisting for more than 6 months. Importantly, there was no difference in ­thrombosis among the patients who ­underwent different surgical ­procedures to treat portal hypertension.

According to the literature, reduced blood flow and ­consequent decreased vein pressure after PVD are associated with ­thrombogenesis^6,7,10-12^. In fact, the purpose of this ­surgical procedure was to reduce the blood pressure in the portal ­system to prevent variceal bleeding^5^. PVD leads to a partial stop in the hepatofugal portal flow to the esophagogastric area. It is ­important to consider that this blockage increases portal flow and pressure in the rest of the portal-venous system^10,21^. The ligature of the splenic artery diminished blood flow to the spleen, and patients in Group 1 showed a reduction in spleen size during the ­post-operative follow-up^12,22,25^.

The etiopathogenesis of the portal system thrombosis is still not adequately understood in the literature^3,6,7^. In most cases, it is transient and recurrent without symptoms. No drugs, such as heparins, salicylic acid, or other ­antithrombotics, have proven effective in preventing or treating specific ­portal ­thrombosis. All drugs have been prescribed based on their systemic effects, which differ from their effects on the ­portal system^10,11^. Considering that portal thrombi are transient and self-limited, the presumed efficacy of these drugs may not align with reality^4,5,11^.

No patient in this series presented total portal ­thrombosis during the follow-up period. All thrombi reduced in size or ­disappeared at different periods, and there was no association between thrombi growth and disappearance with age, sex, or ­surgical procedure. These data are in accordance with the ­literature, which reports that ­partial thrombi occur in most cases without clinical manifestation^13,22^.

In this study, no differences were observed in the ­comparative results of hematological and biochemical exams between patients with and without portal thrombosis. Post-splenectomy ­thrombocytosis was not found to be related to the ­formation of a thrombus in the portal system^4,14,22,25^. Thus, the ­pathophysiology of postoperative thrombosis in patients with schistosomatic portal hypertension remains unknown, despite the numerous studies conducted^1,4,8,13,23,24^.

Patients with splenorenal shunts, another acceptable ­surgical procedure to treat schistosomatic portal hypertension, were not included in this study to maintain the uniformity of this series on PVD. The patients were randomly assigned to each ­surgical ­procedure according to the surgeons' choice in each case. No patient experienced variceal bleeding, and all patients are still alive. Hepatosplenic schistosomiasis is a benign process that does not impair the functions of the liver or spleen. In this study, most of the patients were peasants, and all of them returned to their normal lives after the surgical procedure.

A limitation of this study is the small sample size. Surgical treatment of schistosomiasis is rare, and this study shows the experience of a single institution with a long-term follow-up involving four different types of surgical approaches. Very few studies comparing post-operative portal system ­thrombosis with liver function must be emphasized. Most studies refer only to the presence of portal thrombosis as a frequent ­complication of total splenectomy.

## CONCLUSION

Portal vein thrombosis is a common postoperative ­complication in patients with portal hypertension, ­irrespective of surgical treatment, and it is not associated with patient ­characteristics or any hematological and biochemical tests.

## ETHICAL ASPECTS

This research is part of a series of studies related to portal hypertension treatment. All patients gave their informed consent for inclusion before they participated in the study. The study was conducted in accordance with the Declaration of Helsinki, and the protocol was approved by the Committee of the Research Ethics of the Federal University of Minas Gerais (UFMG), Brazil, registered under the protocol number ETIC 006/08.
